# Obstructive Sleep Apnea: A Marker of Cardiac Remodeling in Patients with Chronic Chagas Disease

**DOI:** 10.5935/abc.20180177

**Published:** 2018-09

**Authors:** Reinaldo B. Bestetti

**Affiliations:** Curso de Medicina - Universidade de Ribeirão Preto, Ribeirão Preto, SP - Brazil

**Keywords:** Chagas Disease, Trypanosoma Cruzi, Chagas Cardiomyopathy, Sleep Apnea, Obstructive, Polisomnography/methods, Atrial Remodeling

Chronic Chagas disease (CCD) continues to be a major scourge for people living in South America and an emergent medical problem outside the American Continent because of world globalization. Chronic Chagas heart disease (CCHD) affects about 30% of patients with CCD, appearing 20-30 years after infection with *Trypanosoma cruzi*.^1^ Prognosis of CCD patients is relentless, with a 5-year mortality approaching 35%.^2^ CCHD patients have an outcome even worse,^2^ particularly those with ventricular and atrial remodeling, which manifests by chronic systolic heart failure and atrial fibrillation.^3,4^

It is, therefore, important to recognize predictors of ventricular and atrial remodeling in patients with CCHD to offer the proper available treatment for patients with this condition. Systolic blood pressure, male sex, and New York Heart Association Functional Class appear to predict ventricular remodeling in patients with CCD.^5^ Conversely, as far as I know, predictors of atrial remodeling have not yet been established for patients with this condition.

In this issue of the Journal, Medeiros et al.^6^ report on an original study of 135 Chagas disease patients (30% of them in the indeterminate form and the remaining with CCHD) who have undergone overnight polysomnography to assess the relationship of sleep-disordered breathing and cardiac remodeling. Importantly, 62% of patients also had concomitant systemic arterial hypertension (SAH). Moderate to severe obstructive sleep apnea (OSA) was found in 21% of patients. Medeiros et al.^6^ confirm that male sex and SAH are predictors of ventricular remodeling, and also discovered that the apnea-hypopnea index, a diagnostic marker of the severity of OAS, was a predictor of both atrial and ventricular remodeling.

The prevalence of OSA is 21% in a general population. It is moderate to severe in 9% of affected individuals,^7^ and increases morbidity and mortality.^8^ OSA has independently been associated with SAH^9^ and with several different types of cardiovascular disorders, including chronic heart failure,^10^ a condition usually associated with cardiac remodeling. It is noteworthy that OSA by itself has also been independently associated with left ventricular remodeling and left atrial diameter.^11^

How can the exciting findings reported by Medeiros et al.^6^ be incorporated in clinical practice? I think that it would be necessary to test the usefulness of polysomnography in patients with CCD without concomitant SAH. By doing that, we could rule out the additive effect of OSA and SAH^11^ on the genesis of cardiac remodeling, as well as to establish the real effect of OSA on the induction of cardiac abnormalities in patients with this condition.

On the other hand, it is important to recognize that a substantial proportion of patients with CCD do not have concomitant SAH in the study by Medeiros et al.^6^ Therefore, it is reasonable to admit that OSA by itself could have induced, at least in part, the atrial and ventricular remodeling observed in that study. The appearance of OSA by itself might represent an additional burden to myocardial function to patients with CCD/CCHD because OSA activates sympathetic activity^12^ and is proinflammatory.^8^

The histological findings observed in catecholamine cardiomyopathy are similar to those found in CCHD,^13^ thus suggesting a role for autonomic dysfunction in the pathogenesis of this disease. Furthermore, proinflammatory cytokines are more increased in patients with CCHD and SAH in comparison with patients with CCHD alone,^14^ suggesting a role for cytokines in the pathogenesis of patients with this condition as well. Clearly, the presence of OSA might represent a potential curable threat for patients with CCHD.

I congratulate Medeiros et al.^6^ for this important study, and I do hope that they continue to pursue this research line not only because its potential contribution to the understanding of the pathogenesis of CCHD, but also for its potential impact on the clinical course of this scourge to our people.
